# Transcriptome and Comparative Chloroplast Genome Analysis of *Vincetoxicum versicolor*: Insights Into Molecular Evolution and Phylogenetic Implication

**DOI:** 10.3389/fgene.2021.602528

**Published:** 2021-03-04

**Authors:** Xiaolei Yu, Wenxiu Wang, Hongxia Yang, Xiaoying Zhang, Dan Wang, Xiaoxuan Tian

**Affiliations:** ^1^State Key Laboratory of Component-based Chinese Medicine, Tianjin University of Traditional Chinese Medicine, Tianjin, China; ^2^Key Laboratory of Pharmacology of Traditional Chinese Medical Formulae, Tianjin University of Traditional Chinese Medicine, Tianjin, China

**Keywords:** *Vincetoxicum versicolor* (Bunge) Decne, transcriptome, chloroplast genome, comparative analysis, phylogeny

## Abstract

*Vincetoxicum versicolor* (Bunge) Decne is the original plant species of the Chinese herbal medicine Cynanchi Atrati Radix et Rhizoma. The lack of information on the transcriptome and chloroplast genome of *V. versicolor* hinders its evolutionary and taxonomic studies. Here, the *V. versicolor* transcriptome and chloroplast genome were assembled and functionally annotated. In addition, the comparative chloroplast genome analysis was conducted between the genera *Vincetoxicum* and *Cynanchum*. A total of 49,801 transcripts were generated, and 20,943 unigenes were obtained from *V. versicolor*. One thousand thirty-two unigenes from *V. versicolor* were classified into 73 functional transcription factor families. The transcription factors bHLH and AP2/ERF were the most significantly abundant, indicating that they should be analyzed carefully in the *V. versicolor* ecological adaptation studies. The chloroplast genomes of *Vincetoxicum* and *Cynanchum* exhibited a typical quadripartite structure with highly conserved gene order and gene content. They shared an analogous codon bias pattern in which the codons of protein-coding genes had a preference for A/U endings. The natural selection pressure predominantly influenced the chloroplast genes. A total of 35 RNA editing sites were detected in the *V. versicolor* chloroplast genome by RNA sequencing (RNA-Seq) data, and one of them restored the start codon in the chloroplast *ndhD* of *V. versicolor*. Phylogenetic trees constructed with protein-coding genes supported the view that *Vincetoxicum* and *Cynanchum* were two distinct genera.

## Introduction

Apocynaceae is a large family of plants distributed globally, which contains around 4,500 species in approximately 370 genera (Endress et al., 2014; Fishbein et al., 2018). *Vincetoxicum versicolor* (also known as *Cynanchum versicolor* in Flora of China) belongs to the Apocynaceae family and is the original plant species of the Chinese herbal medicine Cynanchi Atrati Radix et Rhizoma (Chinese Pharmacopoeia Commission, 2015). However, the genus of this plant has not been unified due to the controversial phylogenetic relationship between the genera *Vincetoxicum* and *Cynanchum*, which may affect the Cynanchi Atrati Radix et Rhizoma application in the world. The phylogenetic relationship between *Vincetoxicum* and *Cynanchum* has been controversial since the first transfer of *Vincetoxicum hirundinaria* and several other Eurasian *Vincetoxicum* species to the genus *Cynanchum* by Persoon in 1805 (Persoon, 1805). Some researchers have suggested that *Vincetoxicum* should be grouped into the genus *Cynanchum* based on the corona structure similarity (Jiang and Li, 1977; Gilbert et al., 1996). On the other hand, these two genera were considered to be distinct, and *Vincetoxicum* was regarded as an independent genus based on molecular data and chemical substances (Qiu et al., 1989; Liede-Schumann, 2000). Besides, the second opinion is supported by studies based on some regions of the nuclear and chloroplast DNA (Yamashiro et al., 2004; Fishbein et al., 2018). Although *Vincetoxicum* is generally considered an independent genus in Apocynaceae taxonomy around the world (Goyder et al., 2012; Endress et al., 2014; Liede-Schumann et al., 2016; Liede-Schumann and Meve, 2018), the concept of *Vincetoxicum* as a section of the genus *Cynanchum* is still reflected in the taxonomy of modern flora in China (Feng et al., 2012; Li et al., 2012; Yang J. et al., 2018). Therefore, more evidence should be provided to promote the unification of the phylogenetic relationship between *Vincetoxicum* and *Cynanchum*.

Chloroplasts originated from ancient endosymbiotic cyanobacteria and are active metabolic centers that sustain life on Earth by converting solar energy into carbohydrates *via* the photosynthesis process and oxygen release (Leister, 2003; Daniell et al., 2016). Chloroplasts carry their own genomes and genetic systems. The typical angiosperm chloroplast genome has a quadripartite structure, with a genome size of 107–218 kb and gene content of 120–130 genes (Daniell et al., 2016; Kim et al., 2019). The chloroplast genome has the characteristics of uniparental inheritance, moderate nucleotide substitution rate, haploid status, and no homologous recombination (Shaw et al., 2005; Hansen et al., 2007; Yang et al., 2019b). These features make it a suitable tool for molecular identification of species and genetic diversity studies (Zhang et al., 2017; Chen et al., 2018). Moreover, the entire chloroplast genome contains more informative sites than chloroplast DNA fragments, which can provide a higher resolution of the phylogenetic relationship at multiple taxonomic levels (Yang X.-Y. et al., 2018). The development of next-generation sequencing technology has led to more and more angiosperm chloroplast genomes available, making comparative chloroplast genomics a convenient and efficient method for phylogenetic and evolutionary studies (Ge et al., 2018; Gu et al., 2019).

Next-generation sequencing not only greatly improves our ability to obtain genomic resources in non-model species but also facilitates the development of the RNA-Seq technique. RNA-Seq is an efficient technology for large scale transcriptome investigations, which provides a convenient way to obtain information from expressed genomic regions quickly and offers an opportunity to solve comparative transcriptomic-level problems for non-model organisms (Logacheva et al., 2011; Zhang et al., 2013). Transcriptome analysis provides an effective way for novel gene discovery (Emrich et al., 2007) and expression profile construction (Fox et al., 2014), as well as for molecular marker development (Zhang et al., 2013) and analysis of adaptive evolution (Jia et al., 2017). As a non-model species, *V. versicolor* lacks transcriptome analysis, delaying molecular studies at the transcriptional level.

RNA editing, which is identified primarily by the RNA-Seq technique, is a repair mechanism derived by species in response to abnormal DNA mutations during evolution. RNA editing is a post-transcriptional process in which the nucleotide in the transcript differs from the encoded DNA sequence by nucleotide insertion, deletion, or conversion (Takenaka et al., 2013). Most RNA editing events occur in internal codons, resulting in amino-acid substitutions. However, in some cases, the ACG codon is restored to the AUG start codon because of the C-to-U RNA editing, contributing to the conservation of the translation start signals at the gene level, which is essential for protein synthesis (Hirose and Sugiura, 1997). This editing-restored start codon has been reported in the chloroplast transcripts from *maize* (*rpl2*), *tobacco* (*psbL*), but especially in the *ndhD* transcript of several species, including *Arabidopsis*, *Betula*, *tobacco*, *spinach*, and *snapdragon* (Neckermann et al., 1994; Wang et al., 2018).

Here, we *de novo* assembled the transcriptome and chloroplast genome of *V. versicolor* and performed a comparative chloroplast genome analysis between species of the genera *Vincetoxicum* and *Cynanchum*. The aims of this study were (1) to characterize the transcriptome and chloroplast genome of *V. versicolor*, (2) to explore the *V. versicolor* molecular evolution, and (3) to provide insights into the phylogenetic relationship between the genera *Vincetoxicum* and *Cynanchum*.

## Materials and Methods

### Plant Materials Collection and DNA and RNA Extraction

The young fresh leaves of a single plant of *V. versicolor* were collected in August 2019 from Tianjin University of Traditional Chinese Medicine (117.06°E, 38.96°N), Tianjin City, China. The voucher specimens were deposited at Tianjin State Key Laboratory of Modern Chinese Medicine, Tianjin University of Traditional Chinese Medicine, Tianjin, China (voucher number 2019bsbq). The collected leaves were snap-frozen in liquid nitrogen and then stored at -80°C until DNA and RNA extraction. The total DNA was extracted using the extract Plant DNA kit (QIAGEN, Germany) following the manufacturer’s instructions. Total RNA was extracted using the QIAGEN RNeasy Plant Mini Kit (QIAGEN, Germany) following the manufacturer’s instructions. The purity and concentration of DNA and RNA were checked using NanoPhotometer^®^spectrophotometer (IMPLEN, CA, United States) and Qubit^®^ DNA Assay Kit in Qubit^®^ 2.0 Fluorometer (Life Technologies, CA, United States), respectively.

### DNA and RNA Sequencing, Assembly, and Annotation of Chloroplast Genome and Transcriptome

The DNA-Seq library with an average insert size of 350 bp was constructed using the Truseq Nano DNA HT Sample Preparation Kit (Illumina United States). The strand-specific RNA-Seq library was constructed using the protocol described by Zhong et al. (2011). Then, the RNA-Seq library was sequenced on the Illumina HiSeqTM 2,500 platform. Subsequently, clean DNA and RNA data were obtained by removing adaptors and low-quality reads from the raw data. The *V. versicolor* chloroplast genome was *de novo* assembled using NOVOPlasty3.7.2 (Dierckxsens et al., 2017). To validate the reads coverage of the assembled chloroplast genome, clean data were mapped to the *V. versicolor* chloroplast genome using bowtie 2 (Langmead and Salzberg, 2012), and the average reads coverage was 2,418×. The *V. versicolor* chloroplast genome was annotated using GeSeq (Tillich et al., 2017), coupled with manual corrections for the start and stop codons. Finally, the *V. versicolor* chloroplast genome was deposited in the National Center for Biotechnology Information (NCBI) GenBank under accession number MT558564. For the transcriptome assembly, high-quality RNA-Seq data were *de novo* assembled into transcripts using Trinity (Grabherr et al., 2011) with min_kmer_cov set to two and other parameters set to default. The trinity-obtained contigs were then linked into transcripts. To remove redundant transcripts and obtain the primary representative of each gene locus, only the longest transcript in each cluster was selected as the unigene for subsequent analysis. Finally, the obtained unigenes were annotated using a BLAST search against the following databases, namely KOG (euKaryotic Ortholog Groups), GO (Gene Ontology), KO (KEGG Ortholog), Swiss-Prot (a manually annotated and reviewed protein sequence database), Nr (NCBI non-redundant protein sequences), Nt (NCBI non-redundant nucleotide sequences), and Pfam (protein family).

### Annotation of Functional Genes, Prediction of Biochemical Pathways, and Detection of Transcription Factors

Gene Ontology functional analysis was implemented using blast2go tool (Götz et al., 2008). The KAAS software (Moriya et al., 2007) was used to predict the biochemical pathways of the *V. versicolor* unigenes based on the KO database. The transcription factors were detected using the iTAK program (Zheng et al., 2016).

### Identification of RNA Editing Sites

RNA-Seq reads were mapped to the chloroplast genome of *V. versicolor* using bowtie 2 (Langmead and Salzberg, 2012). Then, samtools was applied to call single nucleotide polymorphisms to recognize editing sites in the *V. versicolor* chloroplast genome.

### Codon Usage Calculation

The number of codons and the relative synonymous codon usage (RSCU) were calculated using Mega X (Kumar et al., 2018). The effective number of codons (ENc) values against GC content in the third position of synonymously variable codons (GC3s) values of protein-coding genes of chloroplast genome were calculated using CodonW v1.4.4 (Peden, 1999). Then, the relationships between ENc and GC3s were analyzed using the R script.

### Phylogenetic Analyses

A total of 20 chloroplast genomes (Supplementary Table 1) of 18 Apocynaceae species and two Gentianaceae species available in GenBank were collected to reconstruct phylogenetic trees. Besides, another *Vincetoxicum* species (*V. rossicum*) was added to phylogenetic analysis. Although the full-length chloroplast genome of *V. rossicum* was not available, its raw reads were present in NCBI Sequence Read Archive under accession number SRR934046 (Straub et al., 2013). So, a draft chloroplast genome of *V. rossicum* was assembled using NOVOPlasty3.7.2. The draft chloroplast genome was incomplete and contained many degenerate bases in the intergenic regions, but its protein-coding genes were complete and could be used for phylogenetic analysis. The protein-coding genes from 21 chloroplast genomes were extracted, aligned separately, and recombined to construct a matrix using PhyloSuite_v1.1.15 (Zhang et al., 2020). The generated matrix was used to conduct the Bayesian inference (BI) and Maximum likelihood (ML) phylogenies. The BI phylogenies were inferred using MrBayes 3.2.6 (Ronquist et al., 2012) under JC + I + G model, which was determined from the ModelFinder (Kalyaanamoorthy et al., 2017). The ML phylogenies were inferred using IQ-TREE (Nguyen et al., 2015) under an edge-linked partition model for 5,000 ultrafast (Minh et al., 2013) bootstraps, as well as the Shimodaira–Hasegawa-like approximate likelihood-ratio test (Guindon et al., 2010).

## Results and Discussion

### Transcriptome Features

Illumina pair-end sequencing produced 52,502,062 raw reads for *V. versicolor*, and 51,764,112 clean reads were obtained after removing adaptors and low-quality data (Table 1). The base quality value Q20 and Q30 reached 97.51 and 93.01%, respectively, which indicated that the produced data could be used for further analysis. A total of 49,801 transcripts were generated in *V. versicolor*, of which 20,943 unigenes (N50 = 2,128 bp, average length = 1,491 bp) were identified. Most transcripts and unigenes were 1,001–2,000 bp, and the number of transcripts and unigenes over 2,000 bp were 14,787 and 5,443, respectively (Supplementary Figure 1). There were 16,895 unigenes (80.60%) for *V. versicolor* with at least one significant match to the databases discussed earlier and 3,177 unigenes (15.17%) with all significant matches to the databases mentioned earlier (Table 1).

**TABLE 1:** Summary of statistics for the transcriptomes of *V. versicolor*.

Assembly results		Annotation results	
Type	Number	Database	Number (percentage)
Total number of raw reads	52,502,062	KOG	5,344 (25.52%)
Total number of clean reads	51,764,112	GO	12,369 (59.06%)
Q20 of clean data (%)	97.51	KO	6,705 (32.02%)
Q30 of clean data (%)	93.01	Swiss-Prot	13,334 (63.67%)
Total number of transcripts	49,801	NR	16,025 (76.52%)
Total number of unigenes	20,943	NT	11,680 (55.77%)
Min length of unigenes (bp)	301	PFAM	12,369 (59.06%)
Max length of unigenes (bp)	15,605	At least one database	16,895 (80.60%)
N50 of unigenes (bp)	2,128	All	3,177 (15.17%)
Mean length of unigenes (bp)	1,491		

### Gene Ontology and Biochemical Pathways Prediction

**FIGURE 1:**
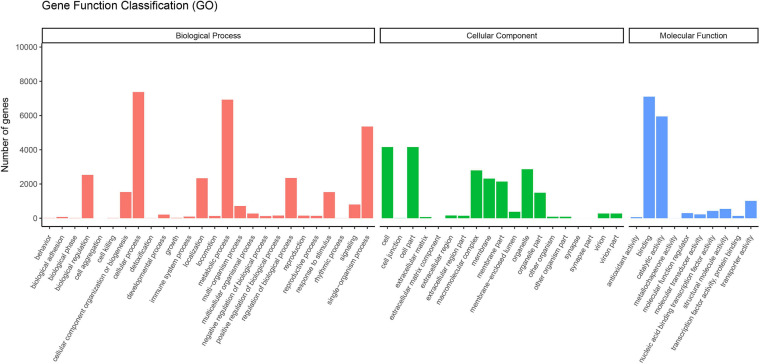
Functional classification of unigenes of *V. versicolor* in GO database. GO terms were annotated according to three main categories “biological processes,” “cellular components,” and “molecular functions.”

The GO concept aims to use a common vocabulary to annotate homologous genes and protein sequences in various organisms in a flexible and dynamic way. Thus, scientists can query and retrieve genes and protein sequences based on their shared biology (Ashburner et al., 2000). The functional classification of unigenes in the GO database was assigned into three categories: biological processes, cellular components, and molecular functions (Figure 1). A total of 12,369 unigenes were assigned to the GO classification groups. In the “Biological processes” group, “Cellular process” (7,374) was the most abundant term. Regarding “Cellular components,” “Cell” (4,159), and “Cell part” (4,159) were the dominant items. In the “Molecular functions” category, “Binding” (7,101) was the largest cluster. Interestingly, the most abundant terms in the corresponding GO categories in *V. versicolor* were highly similar to other angiosperm transcriptomes, such as *Raphanus* (Mei et al., 2016), *Glycyrrhiza* (Jiang et al., 2020), and *Dipteronia* (Zhou et al., 2016). These data suggested that these gene groups are highly expressed and have functional importance in angiosperms.

**FIGURE 2:**
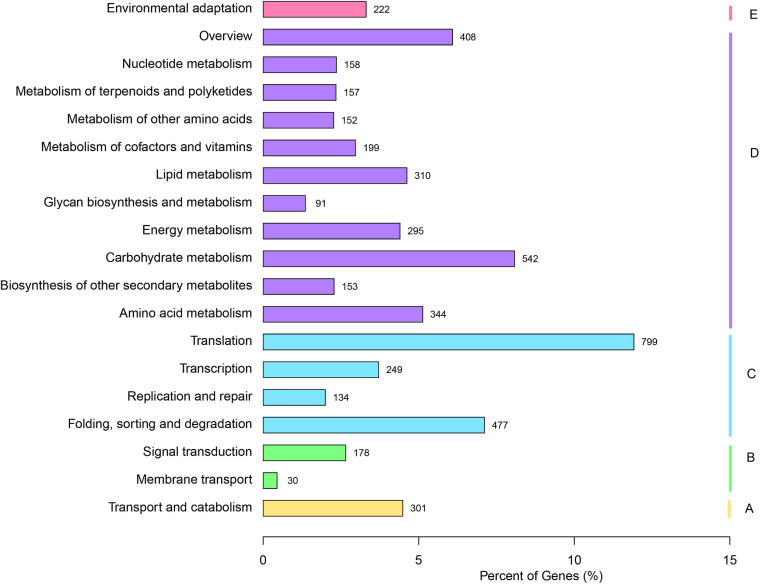
Annotation of unigenes of *V. versicolor* in KO database. “A” presents “Cellular processes,” “B” presents “Environmental information processing,” “C” presents “Genetic information processing,” “D” presents “Metabolism,” and “E” presents “Organismal Systems.”

The KO database is an integrated database resource composed of genes, protein, small molecules, reactions, pathways, diseases, drugs, organisms, and viruses, as well as more conceptual objects, aiming to assign functional meanings to genes and genomes, both at the molecular and higher levels (Kanehisa et al., 2017). For the biochemical pathways prediction in the KO database, a total of 6,705 unigenes were assigned to the KO pathways (Figure 2). The cluster for “Translation” (799) represented the largest group, followed by “Carbohydrate metabolism” (542) and “Folding, sorting and degradation” (477), which indicated that these pathways might be crucial for the *V. versicolor* development.

### Detection of Transcription Factors

Transcription factors play pivotal roles in complex biological processes under multiple environmental signals by regulating the gene transcription through binding to specific DNA sequences in the target gene promoters (Honys and Twell, 2004). Transcription factors are generally classified into different families based on their DNA-binding domains (Jin et al., 2014). A total of 1,032 unigenes *V. versicolor* were classified into 73 functional families (Table 2). Among these families, bHLH transcription factors were the most abundant (57), followed by AP2/ERF (56). It is worth paying attention to these two transcription factor families in the ecological adaptation studies of *V. versicolor*, as they play essential roles in resistance to abiotic stress in plants (Chinnusamy et al., 2003; Yang et al., 2016; Tripathi et al., 2017).

**TABLE 2:** Transcription factor families and corresponding unigenes number identified in *V. versicolor*.

Family	Count	Family	Count	Family	Count	Family	Count	Family	Count
Alfin-like	4	CPP	1	HRT	1	NF	24	STAT	1
AP2/ERF	56	CSD	4	HSF	13	OFP	5	SWI/SNF	17
ARID	8	DBB	2	IWS1	5	Others	36	TAZ	5
AUX/IAA	20	DBP	1	Jumonji	14	PHD	25	TCP	16
B3	27	DDT	6	LIM	2	PLATZ	3	Tify	7
BBR-BPC	5	E2F-DP	5	LOB	9	Pseudo ARR-B	4	TRAF	16
BES1	7	EIL	2	LUG	2	RB	1	Trihelix	22
bHLH	57	FAR1	20	MADS	17	Rcd1-like	2	TUB	9
BSD	1	GARP	25	MBF1	2	RWP-RK	5	ULT	1
bZIP	35	GeBP	6	MED6	1	S1Fa-like	2	VOZ	1
C2C2	46	GNAT	22	MED7	1	SBP	12	Whirly	2
C2H2	46	GRAS	29	mTERF	24	SET	24	WRKY	13
C3H	38	GRF	1	MYB	31	SNF2	26	zf-HD	7
CAMTA	2	HB	37	MYB-related	46	SOH1	1		
Coactivator p15	3	HMG	6	NAC	28	SRS	1		

### Chloroplast Genome Features

**FIGURE 3:**
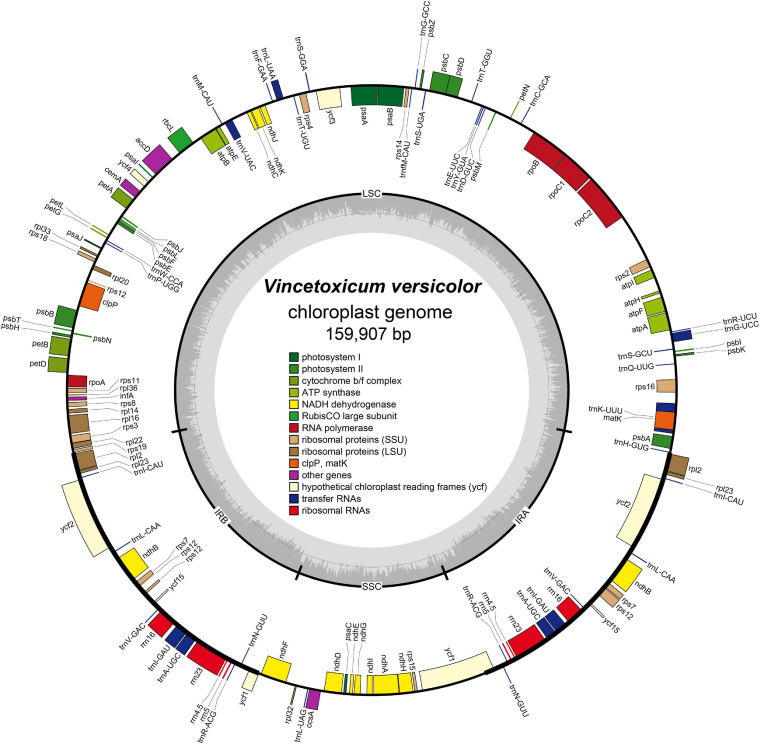
Chloroplast genome map of *V. versicolor*. Genes inside the circle are transcribed clockwise, whereas those on the outside are transcribed counterclockwise. Genes belonging to different functional groups are color-coded. Darker gray in the inner circle represents the GC content, whereas the lighter gray represents the AT content.

The complete chloroplast genome of *V. versicolor* was 159,907 bp in length, including a pair of 24,971-bp IRs separated by 19,456-bp SSC and 90,509-bp LSC regions (Figure 3). This quadripartite structure was a typical feature of the chloroplast genome of most angiosperms (Yu et al., 2019, 2020; Tan et al., 2020). The AT content of the *V. versicolor* chloroplast genome was 62.2%, whereas the AT contents of the LSC, SSC, and IR regions were 63.9, 68.8, and 56.8%, respectively. These data showed that the chloroplast genome exhibited an obvious AT preference and such preference was most evident in the SSC region. The chloroplast genome of *V. versicolor* contained 133 genes, of which 88 were protein-coding genes, 37 were tRNA genes, and 8 were rRNA genes (Table 3). Among these genes, 19 were duplicated in the IR regions, including eight protein-coding genes (*rpl2*, *rpl23*, *ycf2*, *ndhB*, *rps7*, *rps12*, *ycf15*, and *ycf1*), seven tRNA genes (*trnR-ACG*, *trnL-CAA*, *trnV-GAC*, *trnI-CAU*, *trnI-GAU*, *trnA-UGC*, and *trnN-GUU*), and four rRNA genes (*rrn23*, *rrn16*, *rrn5*, and *rrn4.5*). There were 21 genes with introns, and 19 of which (*atpF, petB, petD, ndhA, ndhB* × 2, *rpoC1, rps16, rpl16, rpl2* × 2, *trnK-UUU, trnL-UAA, trnG-GCC, trnV-UAC, trnA-UGC* × 2, *trnI-GAU* × 2) contained one intron, while two genes (*clpP, ycf3*) contained two introns. Although the chloroplast genome sizes of *V. versicolor*, *Vincetoxicum shaanxiense* (NCBI accession number MH210646), *Cynanchum wilfordii* (NC_029459), and *Cynanchum auriculatum* (NC_029460) ranged from 159,907 to 161,241 bp, the gene order, gene content, intron content, and AT content of these genomes were similar (Table 3 and Supplementary Table 2).

**TABLE 3:** Summary statistics of chloroplast genomes of *Vincetoxicum* and *Cynanthum* species.

Genome features	*V. versicolor*	*V. shaanxiense*	*C. wilfordii*	*C. auriculatum*
Genome size (bp)	159,907	160,308	161,241	160,840
LSC size (bp)	90,509	91,335	91,995	91,973
SSC size (bp)	19,456	19,185	19,930	19,667
IR size (bp)	24,971	24,894	24,658	24,600
Number of genes	133	133	133	133
Protein genes [unique]	88 (80)	88 (80)	88 (80)	88 (80)
tRNA genes [unique]	37 (30)	37 (30)	37 (30)	37 (30)
rRNA genes [unique]	8 (4)	8 (4)	8 (4)	8 (4)
Duplicated genes in IRs	19	19	19	19
AT content (%)	62.2	62.2	62.2	62.2
AT content in LSC (%)	63.9	63.9	63.9	63.9
AT content in SSC (%)	68.2	68.1	68.0	68.0
AT content in IRs (%)	56.8	56.7	56.8	56.8

### Detection of Chloroplast RNA Editing Sites

The RNA editing sites in the *V. versicolor* chloroplast genome were identified based on RNA-Seq data. The type and position of the editing sites are shown in Table 4. All RNA editing sites identified were C-to-U. A total of 35 RNA editing sites were detected in the *V. versicolor* chloroplast genome, of which 33 were located in the protein-coding region, and the remaining two were located in the tRNA region (*trnN-GUU*). All identified RNA editing sites occurred at the first and second positions of the codon, resulting in amino acid changes at the transcription level. Among these changes, the change from serine (S) to leucine (L) was the most abundant.

**TABLE 4:** RNA editing sites in the *V. versicolor* chloroplast genome detected by RNA-Seq data.

Gene name	Genome position	Gene position	Codon position	Editing type	Codon change	Amino acid change
*matK*	2,257	1,195	1	C => U	CGG => UGG	R => W
*atpA*	10,941	914	2	C => U	UCA => UUA	S => L
	11,064	791	2	C => U	CCC => CUC	P => L
	11,082	773	2	C => U	UCA => UUA	S => L
*atpI*	15,016	620	2	C => U	UCA => UUA	S => L
*rps2*	16,340	248	2	C => U	UCA => UUA	S => L
	16,454	134	2	C => U	ACA => AUA	T => I
*rpoC2*	21,942	2,840	2	C => U	UCU => UUU	S => F
*rpoB*	28,538	2,426	2	C => U	UCA => UUA	S => L
	30,398	566	2	C => U	UCG => UUG	S => L
	30,413	551	2	C => U	UCA => UUA	S => L
	30,491	473	2	C => U	UCA => UUA	S => L
*rps14*	42,255	149	2	C => U	CCA => CUA	P => L
	42,324	80	2	C => U	UCA => UUA	S => L
*accD*	64,582	1,214	2	C => U	UCG => UUG	S => L
*psaI*	65,640	71	2	C => U	UCU => UUU	S => F
	65,645	76	1	C => U	CAU => UAU	H => Y
*psbE*	70,280	214	1	C => U	CCU => UCU	P => S
*petB*	82,349	611	2	C => U	CCA => CUA	P => L
*rpl23*	92,256	71	2	C => U	UCU => UUU	S => L
	92,274	89	2	C => U	UCA => UUA	S => F
*trnN-GUU*	113,909	39	/	C => U	/	/
*ndhF*	117,658	290	2	C => U	UCA => UUA	S => L
*ndhD*	121,200	1,490	2	C => U	UCU => UUU	S => F
	121,380	1,310	2	C => U	UCA => UUA	S => L
	121,392	1,298	2	C => U	UCA => UUA	S => L
	121,812	878	2	C => U	UCA => UUA	S => L
	122,016	674	2	C => U	UCG => UUG	S => L
	122,091	599	2	C => U	UCA => UUA	S => L
	122,688	2	2	C => U	ACG => AUG	T => M
*ndhG*	123,911	347	2	C => U	CCA => CUA	P => L
*ndhA*	125,380	1,079	2	C => U	UCC => UUC	S => F
*trnN-GUU*	136,508	39	/	C => U	/	/
*rpl23*	158,143	71	2	C => U	UCU => UUU	S => L
	158,161	89	2	C => U	UCA => UUA	S => F

*“Genome position,” “gene position,” and “codon position” refer to the positions of RNA editing in the chloroplast genome, genes, and codons, respectively.*

**FIGURE 4:**
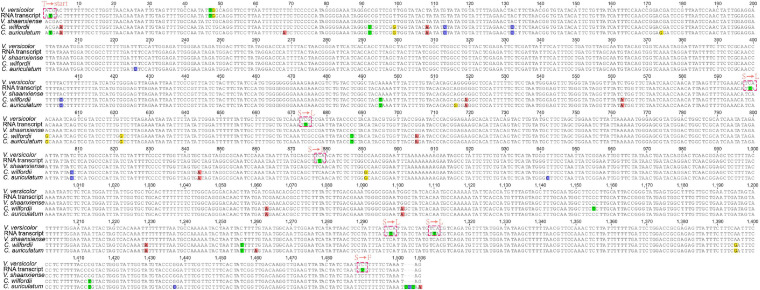
Comparison of chloroplast *ndhD* in *Vincetoxicum* and *Cynanchum* species. Red dotted box represents the amino acid changes at the transcription level. “Start” represents the start codon, whereas “T,” “S,” “L,” and “F” represent threonine, serine, leucine, and phenylalanine, respectively.

We found an interesting phenomenon when checking the annotated genes, in which the chloroplast *ndhD* of *V. versicolor* did not seem to have a start codon at the genome level (the sequence was validated by a polymerase chain reaction and Sanger sequencing data). A further comparison of the chloroplast *ndhD* between species of the genera *Vincetoxicum* and *Cynanchum* showed that only the *C. auriculatum ndhD* started with the standard AUG. In contrast, the *ndhD* of *V. versicolor*, *V. shaanxiense*, and *C. wilfordii* exhibited ACG instead of AUG at the corresponding codon position (Figure 4). Therefore, we speculated that RNA editing restored the start codon AUG in *V. versicolor*, *V. shaanxiense*, and *C. wilfordii*, as observed in *Arabidopsis*, *tobacco*, *spinach*, *Betula*, and *snapdragon* (Neckermann et al., 1994; Wang et al., 2018). Examination of *V. versicolor* transcripts revealed seven RNA editing sites in *ndhD*, one of which appeared on the *ndhD* first codon, causing the codon change from ACG to AUG (this editing site was validated by a reverse transcription-polymerase chain reaction, Supplementary Figure 2). This confirmation of the editing-restored *ndhD* start codon in *V. versicolor* strongly supported our hypothesis despite lacking the transcripts from the other two species.

To further verify whether the editing-restored *ndhD* start codon was a common phenomenon in Apocynaceae, the *ndhD* of 17 Apocynaceae species was compared (Supplementary Table 3). The results showed that almost all of the examined Apocynaceae species exhibited ACG in the first *ndhD* codon (except for *C. auriculatum*), suggesting that the editing-restored *ndhD* start codon was prevalent in Apocynaceae. This kind of editing-restored *ndhD* start codon had also been reported in other angiosperms, especially in dicots (López-Serrano et al., 2001; Tsudzuki et al., 2001). In Apocynaceae, only *C. auriculatum* showed the appropriate AUG start codon in *ndhD*, suggesting that the mutation in this species corrected the start codon of *ndhD* at the genomic level after the interspecific differentiation in *Cynanchum*, as implied by previous studies on Liliaceae and Aloaceae (López-Serrano et al., 2001).

### Codon Usage Analyses

As an important evolutionary feature, the codon usage pattern has been widely investigated in plant chloroplast genomes (Gao et al., 2018; Somaratne et al., 2019; Yang et al., 2019a). To explore the codon usage pattern in the chloroplast genomes of the *Vincetoxicum* and *Cynanchum* species, we calculated the number of codons and RSCU of protein-coding genes in the four chloroplast genomes using Mega X (Supplementary Table 4). The 88 shared protein-coding genes were encoded by 26729, 26671, 26716, and 26586 codons in the chloroplast genomes of *V. versicolor*, *V. shaanxiense*, *C. wilfordii*, and *C. auriculatum*, respectively. AAA encoding lysine was the most commonly used codon in the chloroplast genome of *V. versicolor*, whereas AUU encoding isoleucine was the most abundant codon in the chloroplast genomes of *V. shaanxiense*, *C. wilfordii*, and *C. auriculatum*. In the four chloroplast genomes, the A/U content in the third codon position was 68.70–69.11%, showing the preference for A/U-ending codons. Codon bias contributes to the efficiency of gene expression and, therefore, is generated and maintained by selection pressure (Hershberg and Petrov, 2008). The bias toward A/U in the third codon position is commonly observed in the angiosperm chloroplast genomes (Cui et al., 2019; Mehmood et al., 2020). This reflects the strong selection pressure that affects the codon usage of the chloroplast genome, thus regulating the chloroplast gene expressions. Additionally, except for UUG, all preferred synonymous codons (RSCU > 1) ended with A/U. The usage of the initial codon AUG and tryptophan UGG had no bias (RSCU = 1), as observed in other angiosperms (Li et al., 2019).

**FIGURE 5:**
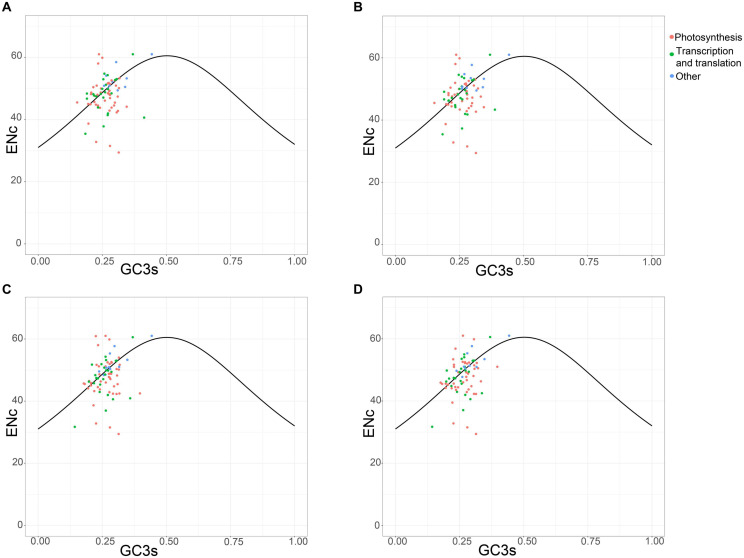
ENc plotted against GC3s based on protein-coding genes of chloroplast genomes of *Vincetoxicum* and *Cynanchum* species. **(A)** *V. versicolor*; **(B)** *V. shaanxiense*; **(C)** *C. wilfordii*; **(D)** *C. auriculatum*.

The plot of the ENc values against the GC3 values is a useful indicator to explore the factors that affect the codon usage. The predicted values are in the expected curve when the codon usage of a gene is constrained only by the G + C mutation bias. Moreover, the predicted values are much lower than the expected curve when natural selection played a major role in optimizing codon usage bias (Wright, 1990). The four chloroplast genomes shared the analogous codon bias pattern (Figure 5). A small number of protein-coding genes followed the standard curve, suggesting that the codon bias of these genes was caused mainly by the nucleotide composition bias in the third codon position. In particular, more than half of the genes were below the curve, indicating that natural selection predominantly influenced these genes. The photosynthesis-related genes represent most of them, revealing their importance so that strong selection pressure is necessary to keep these genes conserved. However, not all photosynthesis-related genes were below the curve. These photosynthesis-related genes exhibited discrete distribution, which implies that other factors such as gene expression level can also affect codon bias (Hershberg and Petrov, 2008).

### Phylogenetic Analysis

**FIGURE 6:**
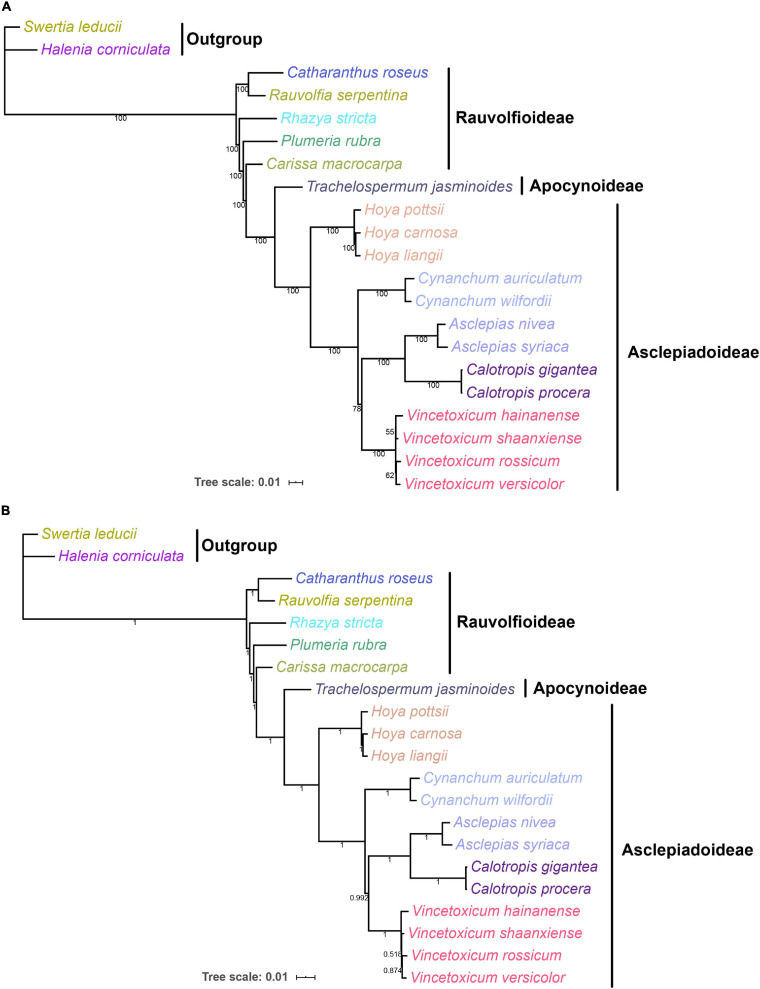
ML and BI phylogenetic trees of Apocynaceae based on 88 protein-coding genes in the chloroplast genome. Numbers below the lines represented ML bootstrap proportions and BI posterior probabilities. *Halenia corniculata* and *Swertia leducii* were set as the outgroups. **(A)** ML tree; **(B)** BI tree.

Complete chloroplast genomes can provide abundant genetic information for understanding the phylogenetic relationships at various taxonomic levels (Huang et al., 2019; Yang et al., 2019b). To explore the phylogenetic relationship between the genera *Vincetoxicum* and *Cynanchum* in the Apocynaceae family, the phylogenetic analysis was conducted based on protein-coding genes of chloroplast genomes of 19 Apocynaceae species (Figure 6). ML and BI trees had a highly similar typology at most branches, except that the position of *Vincetoxicum hainanense* between ML and BI trees was inconsistent. In the ML and BI trees, four *Vincetoxicum* species (*V. versicolor*, *V. shaanxiense*, *V. hainanense*, and *V. rossicum*) were clustered into a monophyletic branch (bootstrap proportions = 100, posterior probabilities = 1), whereas two *Cynanchum* species formed another monophyletic branch (bootstrap proportions = 100, posterior probabilities = 1). Phylogeny between *Vincetoxicum* and *Cynanchum* was described as {*Cynanchum* + [*Vincetoxicum* + (*Asclepias* + *Calotropis*)]}, which strongly supports the previous view (Liede-Schumann, 2000; Yamashiro et al., 2004; Alessandro et al., 2007) that there was no close phylogenetic relationship between the genera *Vincetoxicum* and *Cynanchum*.

## Conclusion

This study was the first effort to characterize the transcriptome and chloroplast genome of *V. versicolor*. A total of 49,801 transcripts were generated, and 20,943 unigenes were obtained from *V. versicolor*. The GO classification showed that “Cellular process,” “Cell,” “Cell part,” and “Binding” were the most abundant terms in the corresponding categories. KO pathway prediction indicated that the “Translation” cluster represented the largest group. A total of 1,032 unigenes from *V. versicolor* were classified into 73 functional transcription factor families. The bHLH and AP2/ERF transcription factors were significantly abundant, suggesting that they should be carefully evaluated in the *V. versicolor* ecological adaptation studies. The comparative analysis showed that the *Vincetoxicum* and *Cynanchum* chloroplast genomes were highly conserved in terms of gene order, gene content, and AT content. They shared an analogous codon bias pattern in which their protein-coding genes exhibited a preference for A/U-ending codons. More than half of the chloroplast genes were predominantly influenced by natural selection pressure, and photosynthesis-related genes accounted for most of them. The RNA-Seq data revealed 35 editing sites in the chloroplast genome of *V. versicolor*, and one of which restored the *ndhD* start codon in *V. versicolor*. Phylogenetic analysis based on ML and BI trees strongly supported the view that *Vincetoxicum* and *Cynanchum* were two distinct genera. Thus, *Vincetoxicum* should be regarded as an independent genus in the Apocynaceae family. Overall, this study provided valuable insights into the evolution and phylogeny of *V. versicolor*.

## Data Availability Statement

The dataset generated for this study can be found in NCBI Sequence Read Archive (SRA) under the accession numbers SRR10838756 (DNA) and SRR10838799 (RNA). The assembled chloroplast genome of *V. versicolor* can be found in GenBank under the accession number MT558564.

## Author Contributions

XT and DW designed the study and revised the manuscript. XY assembled, annotated, analyzed the chloroplast genome and transcriptome, and drafted the manuscript. XY and WW performed the experiment. XY, HY, and XZ analyzed the data. All authors contributed to the article and approved the submitted version.

## Conflict of Interest

The authors declare that the research was conducted in the absence of any commercial or financial relationships that could be construed as a potential conflict of interest.

## Supplementary Material

Supplementary Figure 1 Number and length of transcripts and unigenes of the *V. versicolor* transcriptome.
