# Supplementary material for: Transcriptome and Comparative Chloroplast Genome Analysis of Vincetoxicum versicolor: Insights Into Molecular Evolution and Phylogenetic Implication
Source: Front Genet. 2021 Mar 4;12:602528. doi: 10.3389/fgene.2021.602528 (PMC7970127; doi:10.3389/fgene.2021.602528)
Supplement: Supplementary Figure 1 — Number and length of transcripts and unigenes of the V. versicolor transcriptome. [file Presentation_1.zip › supplement materials/Table S1.docx]

**Table S1. NCBI accession number of chloroplast genomes**

| **Species** | **Accession number** | **Species** | **Accession number** |
| --- | --- | --- | --- |
| *Asclepias nivea* | NC_022431 | *Hoya liangii* | NC_042245 |
| *Asclepias syriaca* | NC_022432 | *Hoya pottsii* | NC_042246 |
| *Calotropis gigantea* | NC_041431 | *Plumeria rubra* | NC_046018 |
| *Calotropis procera* | NC_041440 | *Rauvolfia serpentina* | MN746301 |
| *Carissa macrocarpa* | NC_033354 | *Rhazya stricta* | KJ123753 |
| *Catharanthus roseus* | NC_021423 | *Swertia leducii* | NC_045301 |
| *Cynanchum auriculatum* | NC_029460 | *Trachelospermum jasminoides* | MK783315 |
| *Cynanchum wilfordii* | NC_029459 | *Vincetoxicum shaanxiense* | MH210646 |
| *Halenia corniculata* | NC_042674 | *Vincetoxicum versicolor* | MT558564 |
| *Hoya carnosa* | NC_045868 | *Vincetoxicum hainanense* | MN395661 |
