# Supplementary material for: Transcriptome and Comparative Chloroplast Genome Analysis of Vincetoxicum versicolor: Insights Into Molecular Evolution and Phylogenetic Implication
Source: Front Genet. 2021 Mar 4;12:602528. doi: 10.3389/fgene.2021.602528 (PMC7970127; doi:10.3389/fgene.2021.602528)
Supplement: Supplementary Figure 1 — Number and length of transcripts and unigenes of the V. versicolor transcriptome. [file Presentation_1.zip › supplement materials/Table S2.docx]

**Table S2**. Gene annotation of the *V. versicolor* chloroplast genome.

| **Category** | **Group** | **Genes** |
| --- | --- | --- |
| Photosynthesis related genes | Rubisco | *rbcL* |
|  | Photosystem I | *psaA, psaB, psaC, psaI, psaJ* |
|  | Photosystem II | *psbA, psbB, psbT, psbK, psbI, psbH, psbM, psbN, psbD, psbC, psbZ, psbJ, psbL, psbE, psbF* |
|  | ATP synthase | *atpA, atpB, atpE, atpF*^a^*, atpH, atpI* |
|  | Cytochrome b/f complex | *petA, petB^a^, petD*^a^*, petN, petL, petG* |
|  | Cytochrome csynthesis | *ccsA* |
|  | NADPH dehydrogenase | *ndhA*^a^*, ndhB*^a,c^ (×2)*, ndhC, ndhD, ndhE, ndhF, ndhH, ndhG, ndhJ, ndhK, ndhI* |
| Transcription and translation related genes | Transcription | *rpoA, rpoB, rpoC2, rpoC1*^a^*,* |
|  | Ribosomal proteins | *rps2, rps3, rps4, rps7*^c^ (×2)*, rps8, rps11, rps12*^c^ (×2)*, rps14, rps15*^c^*, rps16*^a^*, rps18, rps19, rpl2*^a, c^ (×2)*, rpl14, rpl16*^a^*, rpl20, rpl22, rpl23*^c^ (×2)*, rpl32, rpl33, rpl36* |
|  | Translation initiation factor | *infA* |
| RNA genes | Ribosomal RNA | *rrn16*^c^ (×2)*, rrn23*^c^ (×2)*, rrn4.5*^c^ (×2)*, rrn5*^c^ (×2) |
|  | Transfer RNA | *trnH-GUG, trnK-UUU*^a^*, trnQ-UUG, trnS-GCU, trnS-UGA, trnS-GGA, trnG-GCC*^a^*, trnR-UCU, trnR-ACG*^c^ (×2)*, trnC-GCA, trnD-GUC, trnY-GUA, trnE-UUC, trnT-UGU, trnfM-CAU, trnL-CAA*^c^ (×2)*, trnL-UAA*^a^*, trnL-UAG, trnF-GAA, trnV-GAC*^c^ (×2)*, trnV-UAC*^a^*, trnM-CAU, trnW-CCA, trnP-UGG, trnI-CAU*^c^ (×2)*, trnI-GAU*^a, c^ (×2)*, trnA-UGC*^a,c^ (×2)*, trnN-GUU*^c^ (×2) |
| Other genes | RNA processing  Carbon metabolism  Fatty acid synthesis  Proteolysis  Conserved open reading frames | *matK*  *cemA*  *accD*  *clpPb*  *ycf1^c^ (×2), ycf15^c^ (×2), ycf2^c^ (×2), ycf3^b^, ycf4* |

^a^—genes with one intron, ^b^—genes with two introns, ^c^—two gene copies in IRs.
