# Supplementary material for: Transcriptome and Comparative Chloroplast Genome Analysis of Vincetoxicum versicolor: Insights Into Molecular Evolution and Phylogenetic Implication
Source: Front Genet. 2021 Mar 4;12:602528. doi: 10.3389/fgene.2021.602528 (PMC7970127; doi:10.3389/fgene.2021.602528)
Supplement: Supplementary Figure 1 — Number and length of transcripts and unigenes of the V. versicolor transcriptome. [file Presentation_1.zip › supplement materials/Table S3.docx]

**Table S3. First codon of *ndhD* in Apocynaceae chloroplast genomes**

| **Species** | **First codon** | **Species** | **First codon** |
| --- | --- | --- | --- |
| *Asclepias nivea* | ACG | *Hoya liangii* | ACG |
| *Asclepias syriaca* | ACG | *Hoya pottsii* | ACG |
| *Calotropis gigantea* | ACG | *Plumeria rubra* | ACG |
| *Calotropis procera* | ACG | *Rauvolfia serpentina* | ACG |
| *Carissa macrocarpa* | ACG | *Rhazya stricta* | ACG |
| *Catharanthus roseus* | ACG | Trachelospermum jasminoides | ACG |
| *Cynanchum auriculatum* | AUG | *Vincetoxicum shaanxiense* | ACG |
| *Cynanchum wilfordii* | ACG | *Vincetoxicum versicolor* | ACG |
| *Hoya carnosa* | ACG |  |  |
