# Supplementary figures and images for: Transcriptome and Comparative Chloroplast Genome Analysis of Vincetoxicum versicolor: Insights Into Molecular Evolution and Phylogenetic Implication
Source: Front Genet. 2021 Mar 4;12:602528. doi: 10.3389/fgene.2021.602528 (PMC7970127; doi:10.3389/fgene.2021.602528)

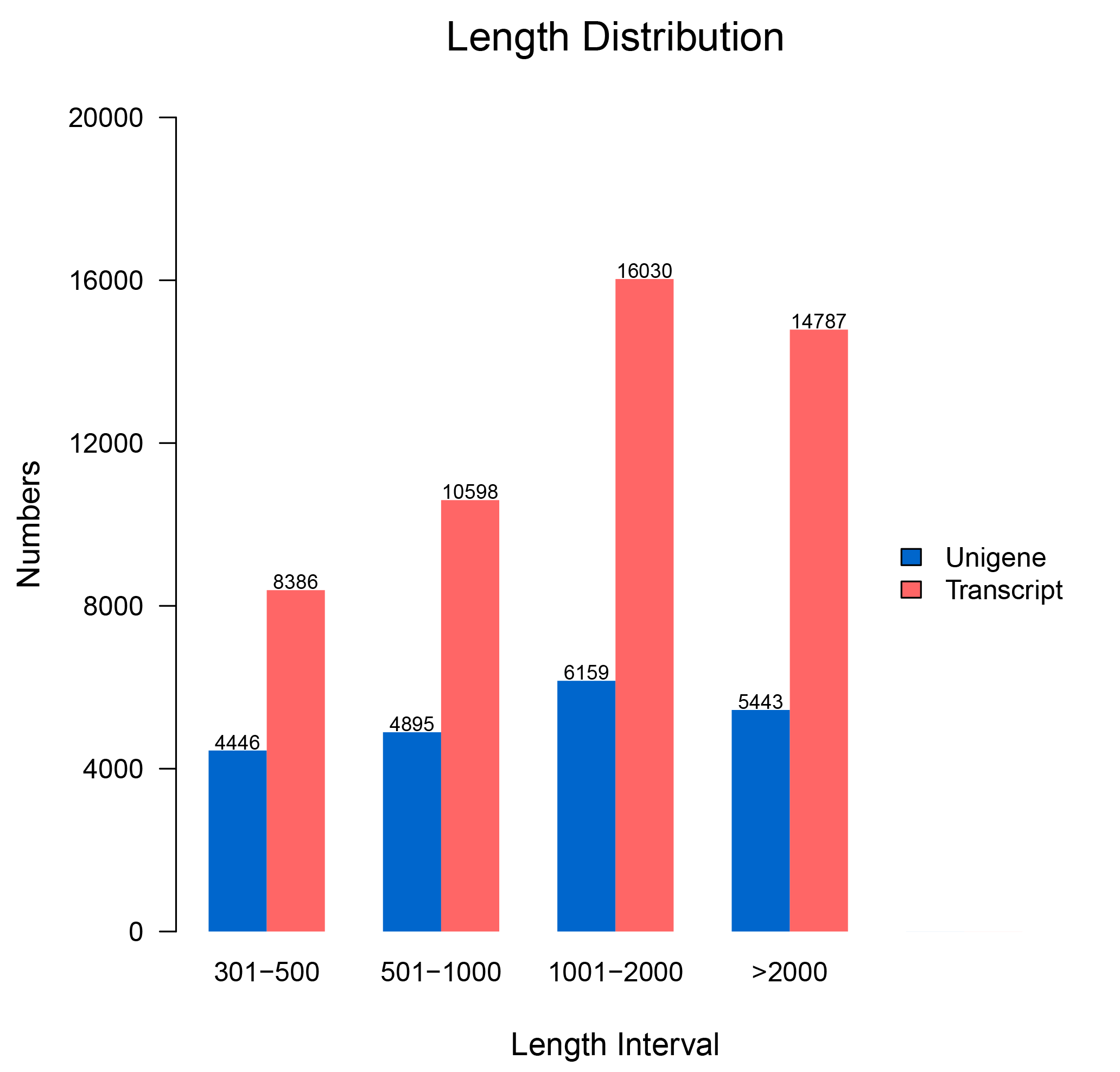

Supplement: Supplementary Figure 1 — Number and length of transcripts and unigenes of the V. versicolor transcriptome. [file Presentation_1.zip › supplement materials/Fig. S1.tif]

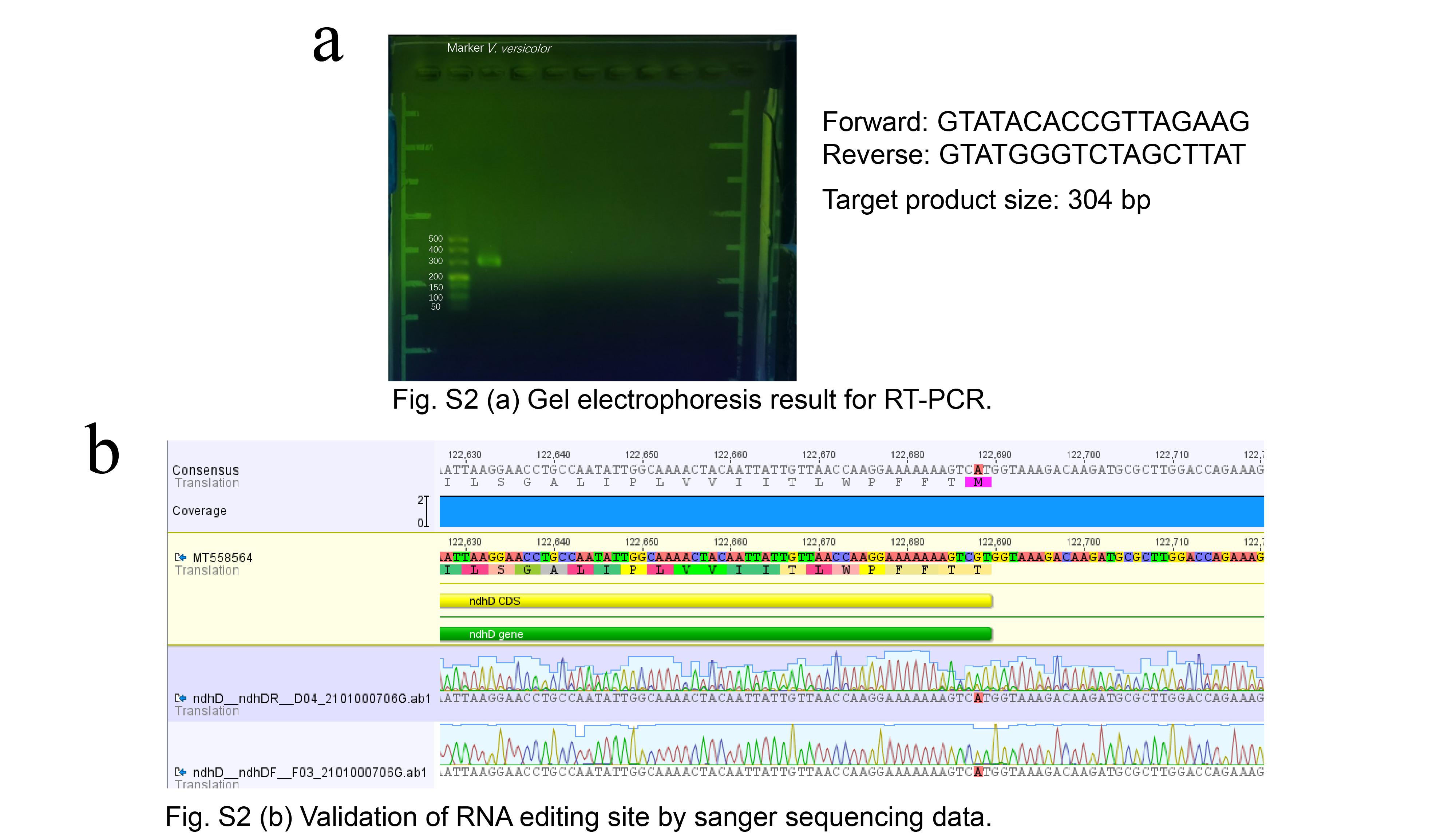

Supplement: Supplementary Figure 1 — Number and length of transcripts and unigenes of the V. versicolor transcriptome. [file Presentation_1.zip › supplement materials/Fig. S2.tif]
